# Total Knee Arthroplasty, All-in-One versus Four-in-One Femoral Cutting Jig System: A Comparison Study

**DOI:** 10.1155/2022/2055537

**Published:** 2022-07-11

**Authors:** Amanda Pratama, Komang Agung Irianto, Rosy Setiawati, Brigita de Vega

**Affiliations:** ^1^Deparment of Orthopedic and Traumatology, Dr. Soetomo General Hospital, Faculty of Medicine, Universitas Airlangga, Surabaya, Indonesia; ^2^Department of Radiology, Dr. Soetomo General Hospital, Faculty of Medicine, Universitas Airlangga, Surabaya, Indonesia; ^3^Institute of Orthopaedics and Musculoskeletal Science, Division of Surgery and Interventional Science, University College London, London, UK

## Abstract

**Background:**

Total knee arthroplasty (TKA) is often indicated for end-stage knee osteoarthritis management. The posterior-stabilized (PS) implant is one of the TKA implants with various component designs, including femoral component cutting jigs. However, little is known about how the differences in cutting jig designs affect the outcomes. This study aims to compare the radiographic and functional outcomes of the patients who underwent cemented TKA using all-in-one and four-in-one femoral component PS implants.

**Methods:**

A retrospective comparative study assessed patients who underwent cemented TKA using PS implants from 2018 to 2019. The patients were divided into all-in-one and four-in-one groups. Demographic data, surgery duration, postoperative radiological findings after one week, and functional outcomes after two years were collected and compared.

**Results:**

A total of 96 patients were included in the study, 55 patients were in all-in-one sample, and 41 patients were in four-in-one sample. The majority of the patients in both groups were female, aged >60 years old, overweight (BMI ≥ 25), and presented with an ASA score of II. We found significantly shorter surgery duration in the all-in-one group compared to the four-in-one group (128.00 ± 36.24 vs. 210.61 ± 57.54, *p*=0.000). The four-in-one group and the all-in-one group showed the insignificant difference in *α*, *β*, *δ*, and *γ* angles (*p*=0.476, 0.273, 0.594, and 0.818). The functional outcomes (SF-12, KSS, and KOOS) showed insignificant differences.

**Conclusion:**

There is no differentiation for the postsurgery functional and radiological outcomes between all-in-one and four-in-one implants.

## 1. Introduction

Knee osteoarthritis (OA) causes gradual functional impairment, stiffness, and pain. It is approximated that over a tenth of people aged 50 years or older are affected globally. In end-stage OA conditions or when all other surgical options fail, total knee arthroplasty (TKA) is indicated as the final option for knee OA management. Moreover, TKA is projected to increase due to the increased aging population in the future. During 2012–2019, there were 1,122,043 TKA procedures performed in the USA; by 2030, it was estimated there would be 1,921,000 TKA procedures performed annually [1]. Despite being considered a highly cost-effective surgery, the literature showed that up to 20% of the patients reported unsatisfactory outcomes [2]. Furthermore, there is little literature on which type of implant provides the best outcomes with the fewest complications.

The surgical technique chosen complied with the globally standardized surgical technique for cemented TKA today, namely, the measured resection, gap balancing, and hybrid technique [3]. In general, there are two types of TKA implants, namely the posterior-stabilized (PS) and cruciate-retaining (CR) systems. The surgeons do not retain both the anterior cruciate ligament (ACL) and the posterior cruciate ligament (PCL) in the PS system. In contrast, the surgeons maintain the PCL in the CR system but not the ACL. Although the continuing debate about the efficacy and superiority of the two systems, a meta-analysis study in 2016 concludes that there is no significant difference between their Knee Society knee Score (KSS), pain score (KSPS), Hospital for Special Surgery score (HSS), kinematic characteristics (postoperative component alignment, posterior tibial slope, and joint-line) and postoperative complication rate. PS system seems to result in a better range of motion (ROM), but still not concluded if that made clinical advantages for postoperative patients [4]. However, the PS implants themselves come with various variations/designs; for instance, the femoral component consists of two designs: the all-in-one (universal) and four-in-one femoral cutting jigs. The all-in-one design only requires one instrument to perform the femoral bone resection, whereas the four-in-one design needs two instruments. The choice of implants from different companies will also determine the instrumentation kit, which may have implications for the operative steps and possibly clinical effectiveness. Thus, our study aims to compare the radiographic (*α*, *β*, *δ*, and *γ* angle) also functional outcomes of the patients who underwent cemented TKA using all-in-one and four-in-one femoral component PS implants. For radiographic angle, the normal value of *α* and *β* angle is 90 ± 3°, 87 ± 3° for *δ* angle, and 3 ± 3° for *γ* angle [5].

## 2. Material and Method

### 2.1. Study Design and Eligibility Criteria

This research is an analytic retrospective study of 96 patients, conducted in Dr. Soetomo General Hospital (41 patients) and Orthopedic Private Hospital (55 patients) in Surabaya, Indonesia. We collected the data of adults suffering from knee osteoarthritis who underwent cemented TKA using the posterior-stabilized system by several Orthopedic Surgeons from General Hospital and Orthopedic Private Hospital at Surabaya between 2018 and 2019. Our inclusion criteria were: (1) adults diagnosed with knee osteoarthritis Kellgren Lawrence grade 3–4, (2) underwent cemented TKA using either all-in-one (Medacta, Switzerland) or four-in-one (Johnson and Johnson, USA), all by posterior-stabilized implant system, and (3) have a minimum of two years of follow-up. We excluded patients with incomplete medical records, those who died within the observation period (two years after surgery), and those who refused to be included in the study. The preoperative score including SF-12, Short Form KSS, and Knee Injury and Osteoarthritis Outcome Score (KOOS) scores, were analyzed for differences and homogeneity in all scoring systems between the preoperative sample of two groups, patient-reported outcome measures (PROMs) evaluated by radiological outcome (*α*, *β*, *δ*, and *γ* angle) and functional outcome (SF-12, Short Form KSS, and KOOS scores).

SF–12 consists of 12 questions to evaluate how patients feel and how well they can do their usual activities. The questionnaire evaluates general patient health, does the patient health give the limitation for moderate activities in daily life, is there any problem for regular activities as a result of the patient physical health, is there any problem for regular activities as a result of the patient's emotional problem, is there any pain symptom that interferes housework and works outside the home, evaluates patient feel and how things have been for some period, and is there any interfere for social activities because of physical health and emotional problem. There will be a numeric score result from SF–12, physical and mental scores. Short-form KSS consist of 6 question to evaluate patient functional outcome: (1) for how long patient can walk before stopping due to knee discomfort, (2–5) how much does the patient knee bother during each activity, such as walking on an uneven surface, climbing or descending stairs, getting up from the low couch or chair without arms, and running activities, and (6) how much does patient knee bother during one discretionary activity. KOOS consists of 5 items to evaluate: pain (evaluate by nine questions to the patient), symptom (evaluate by seven questions to the patient), activities of daily living (evaluate by 17 questions to the patient), sport and recreation function (evaluate by five questions to the patient) and knee-related quality of life (evaluate by four questions to the patient).

### 2.2. Surgical Technique and Approach

The surgical technique performed in both groups was hybrid (combination of measured resection and gap balancing). However, the differences in surgery procedure between the two groups lay in distal femoral bone resection. The all-in-one (universal) group used only one jig to make five cuts (distal femur condyle, anterior cortical bone, posterior condyle, anterior and posterior diagonal) at the distal femur. In contrast, the four-in-one group utilized two cutting jigs: one jig to make four cuts (anterior cortical bone, posterior condyle, anterior and posterior diagonal), and another jig to make the last cut (distal femoral condyle). The surgical approach of these two samples was the medial parapatellar approach.

### 2.3. Data Collection and Assessment

The demographic data (sex, age, and body mass index (BMI)), preoperative ASA (American Society of Anesthesiology) score, surgery duration, postoperative radiological findings, and functional outcomes were recorded. Postoperative radiological findings were evaluated within one week, while the functional outcomes were assessed two years after surgery. The preoperative score (SF-12, Short Form KSS, and KOOS scores) of the two groups were compared by SPSS to ensure that the samples were homogenous.

**Figure 1:**
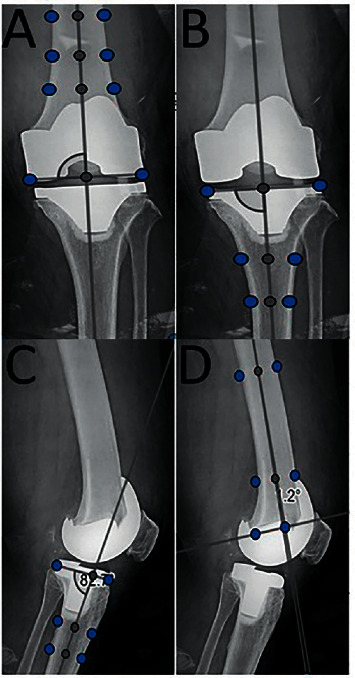
Radiographic outcomes. (a) Alpha (*α*) angle; (b) beta (*β*) angle; (c) delta (*δ*) angle; (d) gamma (*γ*) angle.

The *α*, *β*, *δ*, and *γ* angles of anteroposterior (AP) and lateral views of the knee radiographs in a standing position were measured (Figure 1) [6]: Alpha (*α*) angle is the medial angle between the femoral anatomical axis and a line crossing the domes of the femoral component condyles on the AP radiograph (Figure 1(a)), with a normal range of 90 ± 3°. Beta (*β*) angle is the medial angle between the tibial anatomical axis and a line drawn aligned to the tibial component on the AP radiograph (Figure 1(b)), with a normal range of 90 ± 3°. Delta (*δ*) angle is the posterior angle between the tibial anatomical axis and a line drawn aligned to the tibial component on the lateral radiograph (Figure 1(c)), with a normal range of 87 ± 3°. Gamma (*γ*) angle is the proximal angle between the femoral anatomical axis and a line drawn perpendicular to the femoral component's distal cement interface on the lateral radiograph (Figure 1(d)), with a normal range of 3 ± 3°.

The functional outcomes were evaluated using several PROMs: SF-12 [7], Short Form KSS [8], and KOOS [9], with higher scores indicating better outcomes. However, we could not evaluate the instability and joint motion of KSS because of the coronavirus pandemic; thus, we used the KSS short form [10].

### 2.4. Data Analysis

The demographics and postoperative outcomes of the two groups were compared and analyzed; we also compared preoperative scoring (SF-12, Short Form KSS, and KOOS) between the two groups to ensure that preoperative data between the two samples was used homogenous. Discrete data were presented in frequency and percentage (%), while continuous data were presented in mean and standard deviation (mean ± SD). Discrete data were analyzed using Chi-square or Fisher's test. A normality test for continuous data was performed using the Shapiro-Wilk test. Normally distributed data were analyzed using the independent *t*-test, whereas the abnormally distributed data were analyzed using the Mann-Whitney test. A value of *p* < 0.05 was considered significant. The statistical analyses were performed using SPSS 25.0 (SPSS Inc., Chicago, USA).

## 3. Results

96 patients met the inclusion criteria. The demographic of included patients is presented in Table 1. Overall, the majority of the patients in both groups were female, aged >60 years old, overweight (BMI ≥ 25), and presented with an ASA score of II (with mild systemic disease). After we do statistical analysis, there are no significant demographic profile differences. There is homogeneity from all variances between the two groups (except for the ASA score, and this statistical significance and heterogeneity clinical significance is debatable), which means the samples were homogenous.

The preoperative score comparison (SF-12, Short Form KSS, and KOOS scores) of the two groups is presented in Table 2. We found insignificant differences and homogeneity in all scoring systems between preoperative samples of the two groups. KOOS pain, KOOS quality of life, and KSS running evaluation from two samples have the same score, so there is no value for homogeneity of variances from that variables.

The outcome comparison (surgery duration, radiographic (*X*-ray)) finding and functional outcomes (SF-12, Short Form KSS, and KOOS scores) of the two groups are presented in Table 3. We found significantly shorter surgery duration in the all-in-one (universal) group than in the four-in-one group (*p*=0.000), the other outcome comparisons showed insignificant differences.

## 4. Discussion

The demographics of our patients, who were dominated by overweight females aged >60 years old, were similar across the two groups (Table 1). Previous studies have also reported that TKA is common in females [11, 12] due to the increased risk of osteoarthritis. Female is prone to osteoarthritis because, in advanced age (postmenopausal), the chondroprotective effect of estrogen diminishes as the estrogen level decreases. Increased weight has also been linked to OA due to adipose tissue's increased adipokines (adiponectin and leptin) and proinflammatory cytokines (TNF-*α*, IL-1, and IL-6) production. These adipokines and proinflammatory cytokines induce and enhance the production of matrix metalloproteinases (MMPs) and prostaglandins while inhibiting proteoglycans and collagen type II syntheses. Hence, they are crucial to cartilage matrix degradation in OA pathogenesis [13].

Our study population characteristics showed a significant difference in the ASA score, which is widely used to determine patients' physical status and help to predict operative risks. Although previous studies have revealed that higher ASA scores were associated with more complications and mortalities in general [14], a study by Hooper et al. reported that the mortality rates and functional outcomes (Oxford scores) following TKA in ASA I (completely fit) and II (with mild systemic disease) patients were similar (*p* > 0.05) [15]. As all the included patients were in the range of ASA I-II, the clinical relevance of the statistical difference found is therefore neglectable; thus, our study samples are homogenous and not biased.

TKA procedures always increase every year, making implant companies innovate to simplify the design, make operating procedures easier, and improve cutting accuracy. The all-in-one femoral cutting had created to simplify the surgical procedure and minimize human error during the surgery. This design was also created to reduce surgery duration because the surgeon only needs one jig to make a femoral cut. Based on the previous study by Yasin et al. [16], the result using that implant was satisfactory, but we need to follow up for radiological and functional outcomes.

Our outcome comparison showed a significantly shorter surgery duration in the all-in-one (universal) group compared to the four-in-one group (128.00 ± 36.24 vs. 210.61 ± 57.54 minutes, respectively, *p*=0.000). Several factors for longer surgery duration are grouped into three categories: patient, surgeon, and surgical factors. The patient factors associated with prolonged operative time include advanced patient age, male patients, ASA 3+ (higher degree of comorbidities), obesity, preoperative laboratory findings, and more complex cases [17, 18]. Surgeon factors include surgeon experience (level of training) [17, 19]. Surgical factors such as anesthesia type intraoperative transfusion requirement should also be considered [17, 19]. In this study, we suggest another factor, namely the difference in femoral cutting jigs design (all-in-one/universal vs. four-in-one), as another factor contributing to the surgery duration. The all-in-one femoral cutting jig requires only one instrument for five bone resections at the distal femur. In contrast, the four-in-one needs two instruments that entail additional time to be secured in position.

Previous studies have reported that longer surgery duration was associated with complications, leading to higher revision rates [18, 19]. Interestingly, extensive studies involving national joint registries from New Zealand and the USA showed that not only long TKA surgery duration (>120 minutes and >150 minutes, respectively) was associated with higher revision rates but also the very short ones (<40 minutes and <90 minutes, respectively). While longer procedures lead to more infection and wound dehiscence risks, the very short ones cause more aseptic loosening, which is as detrimental as the formers [20]. Our average operative time was relatively longer than the studies mentioned earlier; this phenomenon seems to be a common finding in developing countries [21], presumably due to the lower TKA volume in developing countries. Improving operating volume from <10 procedures/hospital/year to >200 procedures/hospital/year was associated with an average of 25 minutes shorter operating time in cemented joint replacement surgeries [22].

Nevertheless, literature has reported that the operating time was irrelevant to patients' functional outcomes (assessed by the Oxford score) at six months, five years, and ten years follow-up after TKA surgery [19]. Likewise, our findings showed that the functional outcomes' differences of both groups are insignificant (*p* > 0.05), regardless of the significant surgery duration difference attributable to different femoral component cutting jigs. These insignificant functional outcome findings are expected because the two groups utilized the same prosthesis system, i.e., the PS TKA system. The only difference between the two groups is the femoral cutting jigs. Very few studies compared the different instrument designs in the PS TKA system. Indelli et al. compared different cutting jigs amongst three popular PS knee prostheses (Sigma PS-Johnson and Johnson, Persona-Zimmer, and Vanguard-Biomet). They investigated the maximum volumetric bone resection required for the three different cutting jig designs and found significant differences in the tridimensional PS housing area of the three designs that would result in extra bone resection in certain cutting jig designs [23]. However, to our knowledge, there is no study comparing the effects of femoral cutting jig design differences on the outcomes.

Our study has several limitations. Firstly, we could not evaluate the instability and joint motion as a part of KSS because of the coronavirus pandemic. However, the KSS instrument used was the KSS short form, which has also been validated [10]. Secondly, there might be a performance bias for surgery duration time because the patients were operated on by different surgeons. Because of the limitation of the patient sample, we cannot control these confounding factors, and we do not have a huge number of patients post-TKA that operated on by the same surgeon. Future studies should compare the postoperative ROM joint stability with a larger sample and evaluate postoperative readmissions, reoperations also infections rate.

## 5. Conclusion

There is no differentiation for the postsurgery functional and radiological outcome between all-in-one and four-in-one TKA cutting jig system.

## Figures and Tables

**Table 1:** Demographics of the patients.

Parameter	All-in-one (*n* = 55)	Four-in-one (*n* = 41)	*p* value	Homogeneity	Method
Sex	Male: 12 (12.5%)	Male: 10 (10.4%)	0.767	0.561^*∗∗*^	Chi-square test
	Female: 43 (44.8%)	Female: 31 (32.3%)			
Age (years)	65.69 ± 8.01	62.83 ± 7.96	0.086	0.583^*∗∗*^	Independent *t*-test
BMI (kg/m^2^)	27.80 ± 4.66	28.00 ± 3.46	0.676	0.246^*∗∗*^	Mann-Whitney test
ASA score	ASA I: 7 (7.3%)	ASA I: 12 (12.5%)	0.044^*∗*^	0.000	Chi-square test
	ASA II: 48 (50%)	ASA II: 29 (30.2%)			

*Note.* ^*∗*^Statistically significant (*p* < 0.05). ^*∗∗*^Statistically homogenous (*p* ≥ 0.05).

**Table 2:** Preoperative score comparison between all-in-one and four-in-one implant.

Parameter		All-in-one (*n* = 55)	Four-in-one (*n* = 41)	*p* value	Homogeneity of variances
SF-12	Physical score	20.54 ± 0.64	20.53 ± 0.65	0.609	0.719^*∗∗*^
	Mental score	61.49 ± 2.25	60.96 ± 2.26	0.254	0.727^*∗∗*^
KSS (short form)	How long can you walk (0–20)	9.82 ± 2.01	10.05 ± 2.01	0.578	0.602^*∗∗*^
	Walking on an uneven surface (0–15)	7.64 ± 1.50	7.46 ± 1.51	0.578	0.602^*∗∗*^
	Climbing or descending stairs (0–15)	7.36 ± 1.50	7.54 ± 1.52	0.578	0.602^*∗∗*^
	Getting up from a low couch or chair without arms (0–15)	7.64 ± 1.50	7.46 ± 1.51	0.578	0.602^*∗∗*^
	Running (0–20)	8.00 ± 0.00	8.00 ± 0.00	1.000	
	Discretionary activity (0–15)	7.36 ± 1.50	7.54 ± 1.51	0.578	0.602^*∗∗*^
	Total (0–100)	47.81 ± 2.01	48.05 ± 2.02	0.578	0.602^*∗∗*^
KOOS	Pain	56.00 ± 0.00	56.00 ± 0.00	1.000	
	Symptoms	48.18 ± 2.01	47.95 ± 2.04	0.578	0.602^*∗∗*^
	Activities of daily living (ADL)	56.45 ± 0.50	56.51 ± 0.50	0.578	0.602^*∗∗*^
	Sport and recreation	47.73 ± 2.51	47.44 ± 2.53	0.578	0.602^*∗∗*^
	Quality of life	44.00 ± 0.00	44.00 ± 0.00	1.000	

*Note.* All tests were analyzed using Mann-Whitney tests. ^*∗*^Statistically significant (*p* < 0.05). ^*∗∗*^ Statistically homogenous (*p* ≥ 0.05).

**Table 3:** Outcome comparison between all-in-one & four-in-one implant.

Parameter		All-in-one (*n* = 55)	Four-in-one (*n* = 41)	*p*-value
Surgery duration (minutes)		128.00 ± 36.24	210.61 ± 57.54	0.000^*∗*^
*X*-ray finding	Alpha (*α*) angle^1^	97.02 ± 2.77	96.35 ± 5.46	0.476
	Beta (*β*) angle^1^	86.62 ± 2.80	86.03 ± 2.41	0.273
	Delta (*δ*) angle^1^	86.33 ± 4.36	86.81 ± 4.24	0.594
	Gamma (*γ*) angle	7.61 ± 5.47	7.38 ± 4.56	0.818
SF-12	Physical score	51.79 ± 7.99	51.58 ± 7.96	0.119
	Mental score	58.10 ± 0.72	58.06 ± 0.56	0.815
KSS (short form)	How long can you walk (0–20)	17.82 ± 2.96	16.88 ± 2.76	0.059
	Walking on an uneven surface (0–15)	14.78 ± 0.96	15.00 ± 0.00	0.131
	Climbing or descending stairs (0–15)	13.91 ± 3.18	11.93 ± 5.71	0.100
	Getting up from a low couch or chair without arms (0–15)	14.56 ± 2.19	14.49 ± 2.41	0.724
	Running (0–20)	1.04 ± 2.96	1.61 ± 4.09	0.676
	Discretionary activity (0–15)	14.56 ± 2.12	15.00 ± 0.00	0.079
	Total (0–100)	76.67 ± 7.98	74.90 ± 8.07	0.063
KOOS	Pain	97.20 ± 5.05	98.98 ± 1.59	0.082
	Symptoms	79.07 ± 8.63	80.76 ± 5.33	0.184
	Activities of daily living (ADL)	97.31 ± 5.51	98.34 ± 1.76	0.494
	Sport and recreation	69.87 ± 16.83	72.80 ± 8.37	0.736
	Quality of life	88.87 ± 12.26	92.93 ± 3.16	0.178

*Note.* All tests were analyzed using Mann-Whitney tests unless stated otherwise. 1, analyzed using an independent *t*-test. ^*∗*^Statistically significant (*p* < 0.05).

## Data Availability

The data used to support the findings of this study are included in the article.
